# Helicobacter pylori prevalence and clinical significance in patients with quiescent Crohn’s disease

**DOI:** 10.1186/s12876-017-0588-7

**Published:** 2017-02-13

**Authors:** Adi Lahat, Uri Kopylov, Sandra Neuman, Nina Levhar, Doron Yablecovitch, Benjamin Avidan, Batia Weiss, Shomron Ben-Horin, Rami Eliakim, on behalf of the Israeli IBD research Network (IIRN), Iris Dotan, Henit Yanai, Yehuda Chowers, Marianne M. Amitai

**Affiliations:** 1 Department of Gastroenterology, Sheba Medical Center, Tel Hashomer, Tel Hashomer, Israel; 2 Sackler School of Medicine, Tel Aviv University, Tel Aviv, Israel; 3 Edmond and Lily Safra Children’s Hospital, Tel Hashomer, Israel

**Keywords:** Crohn’s disease, Helicobacter pylori, Video capsule endoscopy, Prevalence, Eradication

## Abstract

**Background:**

Helicobacter pylori (HP) infection is present in about 50% of the global population, and is associated with chronic gastritis, peptic disease and gastric malignancies. HP prevalence in Crohn’s disease (CD) patients was shown to be low compared to the general population, and its influence on disease activity is yet to be determined. Our aims were to determine the prevalence of HP in a selected group of CD patients with quiescent disease, and to assess the influence of its eradication on disease activity and endoscopic and laboratory activity measures.

**Methods:**

Consecutive CD patients with quiescent disease underwent meticulous disease evaluation with MR enterography (MRE), video capsule endoscopy (VCE), CRP, fecal calprotectin and CDAI. All patients were tested for the presence of HP using stool antigen detection kit. Patients infected with HP were offered eradication treatment with sequential therapy. HP eradication was confirmed using urease breath test and stool antigen test. The influence of HP eradication on disease activity was assessed.

**Results:**

Out of 56 patients enrolled, six patients (10.7%) had HP infection. Of them, five patients had gastro- duodenitis per VCE. All HP positive patients were offered eradication treatment and underwent successful eradication. Notably, 23 (50%) of patients had proximal disease per VCE, most of them (78%) were HP negative.

CDAI, CRP, fecal calprotectin and VCE Lewis inflammatory score did not change significantly following HP eradication, Gastric findings on VCE were not impacted by HP eradication.

**Conclusions:**

The prevalence of HP infection in patients with quiescent CD is relatively low. Eradication of the bacteria did not significantly change neither disease activity measures nor the presence of gastro- duodenitis per VCE, suggesting it might be part of proximal CD. The influence of HP on CD activity merits further investigation.

## Background

Helicobacter pylori (HP) infection is one of the most prevailing global pathogens in humans, and can be detected in 50% of the world’s population [1]. Its prevalence varies considerably in association by geography, ethnicity, age, and socioeconomic factors.

This Gram- negative bacterium causes chronic inflammation in the gastric mucosa, which leads to atrophic and metaplastic changes. Thus, HP infection is associated with chronic gastritis, peptic disease and gastric malignancies [2, 3]. Crohn’s disease (CD) is a chronic inflammatory condition that can affect the gastrointestinal system from the mouth to the anus. Inflammation is transmural, and therefore may be cause internal abscesses, fistula between adjacent organs, spontaneous viscous perforations and fibrotic strictures.. The disease may cause significant morbidity and diminished life quality [4–8]. Disease behavior is characterized by periods of flare-ups with active symptomatic disease and periods of remission [9]. Over the years, many epidemiological studies showed low incidence of HP infection in patients with CD [10–18].

A recent meta-analysis demonstrated negative association between HP infection and CD with OR of 0.38 (95% CI 0.31 to 0.47, *p* value <1e-10) [19].

The reason for that negative association is yet to be determined. One hypothesis suggests that medications used for CD treatment may eradicate HP. Another suggestion along the same line is that CD mucosal alterations might prevent HP colonization [14, 15, 18–22]. An alternative argument suggests a protective effect for HP infection from the occurrence of CD.

CD is categorized by selectively activation of type 1 T helper lymphocyte (Th1) and Th17-related cytokines involved in innate immunity (interleukin (IL) 12, IL-23, IL-27) [23]. HP was shown to produce IL-18 which causes accumulation of tolerogenic dendritic cells and highly suppressive regulatory T cells (Tregs) that help suppress the inflammatory process [24].

High frequency of gastritis and duodenitis was described in CD patients with no association to HP infection [20–22]. However, data assessing the effect of HP infection and eradication on proximal disease and on disease activity in patients with CD is scarce.

Thus, in our study we focused on patients with known quiescent Crohn’s disease that were prospectively evaluated for disease activity measures before and after HP eradication.

## Methods

### Patient population

Study population consisted of adult (>18 years) CD patients with known small bowel Crohn’s disease in clinical remission, as determined by the validated Crohn’s disease activity index (CDAI) of <150, or patients suffering from mild disease symptoms, presented by CDAI of 150–220. All patients included were treated with stable medication doses and on corticosteroid-free remission for 3–24 months. Patients maintained on constant treatment throughout study duration.

Patients that were unable to provide informed consent were excluded from the study. Patients with severe comorbidities or with any condition that will prevent them from swallowing VCE (difficulty in swallowing, history of aspirations or dysphagia, known or suspected intestinal obstruction or severe bowel stricturing) or undergoing MRE (claustrophobia or implanted metal objects or cardiac pacemaker) were excluded as well.

### Imaging studies

All patients underwent an MRE upon enrollment. MR image acquisition was performed using a previously described protocol [25]. A patency capsule (PC) test was performed in all patients with active small bowel disease detected on MRE. If the PC was not expelled from the small bowel within 30 h, the patient was withdrawn from the study. Mucosal inflammation was quantified using the Lewis inflammatory score (LS). Active inflammation was defined as a segmental Lewis score of ≥135 [26]. A board-certified gastroenterologist with over 10 years of experience in capsule endoscopy read the capsule videos. Gastric and duodenal findings were described for each study.

### Inflammatory biomarkers and disease activity measures

Fecal calprotectin, CRP and complete blood count (CBC) were measured routinely every 3 months. Fecal calprotectin levels were measured using the Quantum blue calprotectin kit (BÜHLMANN Laboratories AG, Basel, Switzerland). The reported value range is 30 to 300 μg/g. Levels above 100 μg/g were considered positive. CRP levels were considered elevated if > 5 mg/l.

Patients underwent physician’s evaluation and CDAI assessment for disease activity every 3 months.

### HP assessment and treatment

All patients were tested for the presence of HP using stool antigen detection kit as part (CerTest H. pylori one step card test, Certest Biotec S.L. Zaragoza, Spain). Stool antigen test was chosen for HP detection since this method is noninvasive and most importantly is not influenced by antibiotics or Proton Pump Inhibitors (PPI) treatment. This kit has a sensitivity of >94%, pecificity of >99% and Positive predictive value (PPV) of >99% for HP detection. Patients infected with HP were offered eradication treatment with sequential therapy consisted of 5 days of esomeprazole (40 mg) and amoxicillin (1000 mg) twice daily, followed by 5 days of esomeprazole (40 mg), clarithromycin (500 mg) and Tinidazole (500 mg) twice daily [27]. Notably, sequential therapy is the standard of care for patients infected with HP in our institution. Prior to eradication treatment patients received detailed explanation regarding HP infection implications and the importance of compliance and adherence to treatment. HP eradication was confirmed using urease breath test and stool antigen detection kit 4 weeks following eradication treatment completion.

The impact of HP eradication on disease activity and degree of proximal small bowel inflammation was assessed using the disease activity measures specified above, 8 weeks after eradication conformation.

### Statistical analysis

Descriptive statistics were presented as means ± standard deviations for continuous variables and percentages for categorical variables. Categorical variables were analyzed by Chi Square/Fisher’s exact test and continuous variables-by *T*-test/Mann Whitney test, as appropriate. *P* < 0.05 was considered significant. All computations were performed with the MedCalc Software (Marieke, Belgium).

## Results

**Fig. 1:**
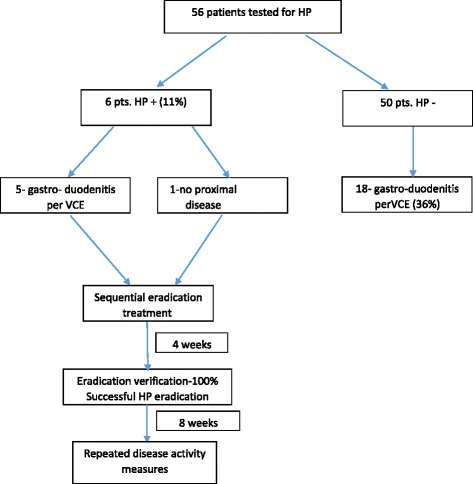
Study design

Fifty six patients were included in the study. Patients’ demographic data and disease characteristics are shown in Table 1. None of the patients was tested for HP prior to this study. A schematic graph of study design is shown in Fig. 1.

**Table 1:** Patients’ demographic data and disease characteristics

		Number	Percent
Male/female		30/26	53.6/46.4
Age at diagnosis (years)		26 ± 11	
Disease duration (years)		6 ± 5	
Clinical remission		52	92.3
Smoking status	current	11	19.6
	never smoked	36	64.3
	past smoking	9	16.1
Previous surgery		9	16.1
Perianal disease		13	23.2
Current medical treatment	Thiopurine	25	44.6
	Anti-TNF	21	37.5
	Combined anti-TNF + thiopurine	7	12.5
No medical treatment		11	19.6

Out of all patients included, only six patients (10.7%) were infected with HP. Of them, five patients had gastro- duodenitis per VCE and one had no proximal disease and no upper GI symptoms. All HP positive patients were offered eradication treatment with sequential therapy detailed above. All infected patients agreed to receive eradication treatment, and all treated patients underwent successful eradication, as was confirmed by a negative urease test and negative stool antigen test. Thus, a 100% eradication rate was achieved.

Notably, 23 (50%) of patients had proximal disease per VCE, most of them (78%) were HP negative.

Four weeks after a verified eradication, a repeated evaluation of disease activity was performed. Physical examination, CBC, CRP was performed and CDAI was calculated. Capsule endoscopy was ingested as well 12 weeks following eradication. Disease activity measures before and 12 weeks following HP eradication in all patients treated was compared, and are shown in Table 2. A separate analysis was performed only for patients who had gastro-duodenitis per VCE before HP eradication. Results are shown in Table 3.

**Table 2:** Disease activity measures in all HP positive patients before and after eradication (*n* = 6)

Disease measures	Before eradication	After eradication	p
CDAI	27.5 ± 21.25	22.83 ± 28.25	NS
CRP	1.17 ± 0.82	2.97 ± 4.7	NS
Fecal calprotectin	75 ± 79.37	112.17 ± 123.2	NS
Lewis Score	425 ± 334.29	564.83 ± 529.46	NS

*NS* non-significant

**Table 3:** Disease activity measures in HP positive patients with gastroduodenitis on VCE before and after eradication (*n* = 5)

Disease measures	Before eradication	After eradication	p
CDAI	23.18 ± 25.6	30.91 ± 25.2	NS
CRP	1.218 ± 0.9	5.12 ± 3.398	NS
Fecal calprotectin	85 ± 85.25	74.6 ± 71.57	NS
Lewis Score	357.33 ± 465	587.995 ± 589.8	NS

*NS* non-significant

As shown in Tables 2 and 3, all disease activity measures as well as anatomical disease distribution (measured by VCE Lewis inflammatory score) were unchanged following HP eradication. Hence, HP eradication had no influence on disease activity.

**Fig. 2:**
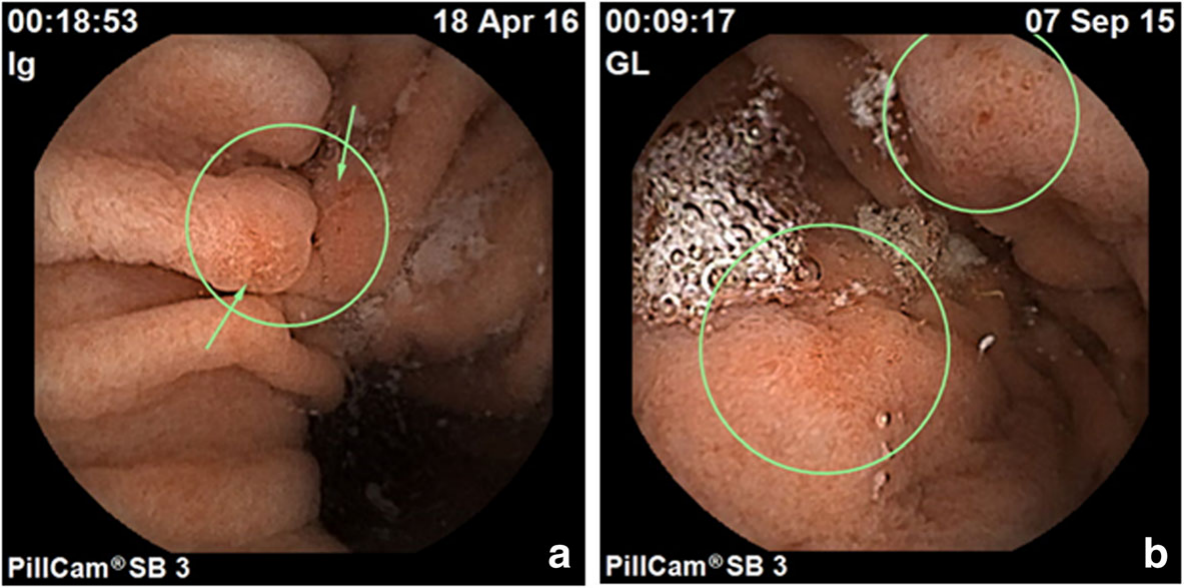
Stomach inflammatory changes by VCE. **a** Gastric erosions (*circled*) prior to HP eradication. **b** Gastric erosions (*circled*) post HP eradication

**Fig. 3:**
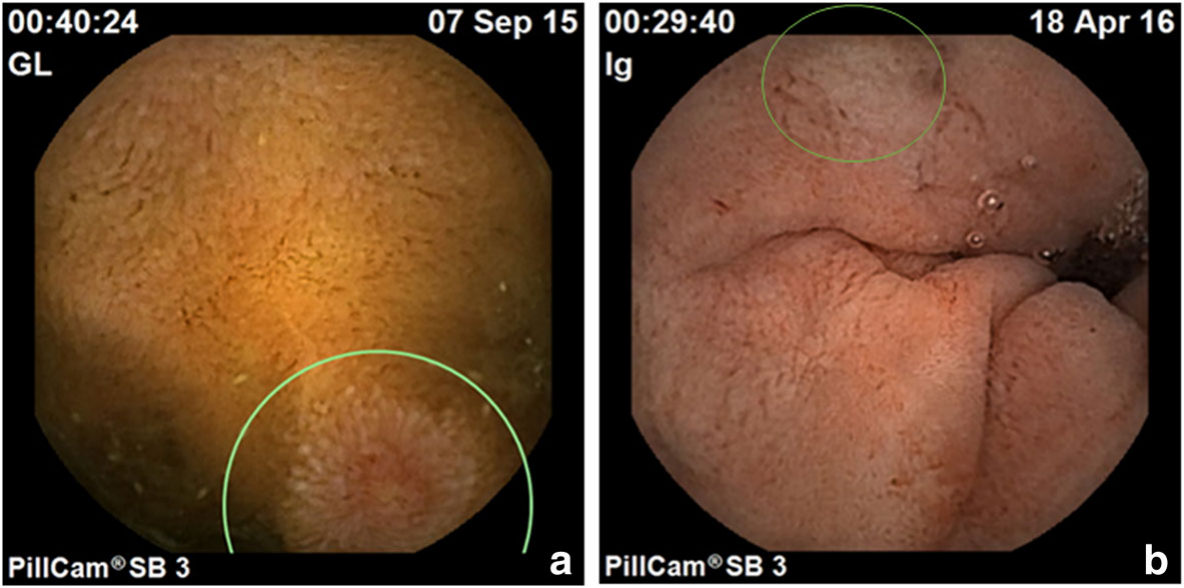
Duodenum inflammatory changes by VCE0. **a** Erosion of proximal duodenum (*circled*) Prior to HP eradication. **b** Erosions in proximal duodenum (*circled*) Post HP eradication

Figures 2a, b and 3a, b show active CD in the proximal digestive system demonstrated by mucosal erosions in stomach and duodenum - unchanged before and after eradication treatment, respectively.

## Discussion

HP infection is one of the most prevalent infectious diseases worldwide. However, in CD patients its prevalence was shown to be lower than in general population [10–18]. The reason for that remains to be determined, as is the mutual impact of CD and HP infection on patients’ symptoms, severity of the disease and on disease distribution. In the current study, we initially assessed the prevalence of HP infection in a homogenous group of CD patients with quiescent disease, and then tried to assess prospectively the impact of HP eradication on disease activity measures and on anatomical inflammatory distribution.

In agreement with data in the literature [10–18], HP infection rate was low among our patients’ group, with only 11% of patients tested positive to HP. The current prevalence of HP infection in Israel is unknown, yet data from 2002 [28] suggest a 60% prevalence among the general population in Israel. Thus, even if we assume a possible reduction in HP infection in the general population during the last decade, HP infection among CD patients is still significantly low. Notably, none of our patients was tested for the presence of HP before entering the study, and consequently none was ever treated for HP eradication. Therefore, we believe that our data reflects the true prevalence of HP infection in CD patients.

All the patients who tested positive for HP were offered eradication treatment, though one of them was a-symptomatic. Since HP is a well known carcinogen with a strong proven relation to gastric malignancy [2, 3], we decided to offer eradication treatment to all infected patients.

In order to achieve maximal eradication rates patients were treated with a 10 day sequential therapy, which was shown to have eradication success of up to 94% [29].

In addition, at treatment initiation patients received individual consultation with emphasis on the importance of compliance and adherence to the offered treatment. Eradication was verified both with urease breath test and with stool antigen detection kit. Using this meticulous treatment option, we managed to reach a 100% eradication rate.

Obviously, since our treatment group consisted of 6 patients only, the results are underpowered to assess treatments’ efficacy. However, we believe that the combination of a high- efficacy eradication protocol and highly motivated patients with high compliance rates probably lead to this unusually high eradication rate.

Eradication was verified using breath test and stool antigen kit 4 weeks following treatment completion [30].

Eight weeks following eradication verification, patients underwent repeated disease evaluation, including VCE. Data in literature support repeated endoscopy for peptic disease 4 weeks following HP eradication treatment [31]. Therefore, we postulated that 8 weeks following eradication verification and 12 weeks following end of eradication treatment is appropriate for reevaluation, assuming that all mucosal damage caused by HP would be healed.

Our results show no improvement in proximal disease or in disease measures following HP eradication. Hence, CDAI, CRP and fecal calprotectin levels did not differ significantly after HP eradication, implicating disease activity was unchanged following eradication. Moreover, the VCE Lewis inflammatory score, as a measure of disease activity and distribution was showed no statistically significant improvement following HP eradication as well. These results were unchanged after separate sub analysis of patients with gastro-duodenitis per VCE.

These data implicates that the proximal disease as well as disease activity was unrelated to HP. To the best of our knowledge, up to date, no study addressed disease activity measures pre versus post HP eradication in CD patients.

In our study, 50% of patients had gastro-duodenitis per VCE. These results are in agreement with previous studies that showed an increased prevalence of gastro- duodenitis disease in CD patients with no association to HP infection [20–22].

Our study has several limitations. First, our cohort of patients was relatively small. Therefore, due to the low prevalence of HP infection in CD patients, the number of HP infected patients was very low. The small number of patients included might affect the applicability of our results.

Another theoretical drawback of our study is a selection bias- only patients with quiescent disease were included, thus patients with active CD might have had different results.

However, we feel that our data supports the evidence showing high incidence of gastroduodenitis in patients with quiescent CD, unrelated to HP infection.

## Conclusions

HP infection in patients with quiescent CD is lower than in the general population, and reaches 11%. Eradication of the bacteria did not change significantly neither disease measures nor the presence of gastro- duodenitis per VCE, suggesting it might be part of proximal CD. The influence of HP on CD activity merits further investigation.

## Abbreviations

CBC: Complete blood count
CD: Crohns’ disease
CDAI: Crohn’s disease activity index
HP: Helicobacter pylori
IL: Interleukin
LS: Lewis inflammatory score
MRE: MR enterography
PC: Patency capsule
PPI: Proton pump Inhibitor
Th1: Type 1 T helper lymphocyte
VCE: Video capsule endoscopy
